# The Impact of Organic Bentonite Content on the Properties of Stereolithographic 3D-Printed Silicon-Based Ceramic Core Paste

**DOI:** 10.3390/ma18081855

**Published:** 2025-04-18

**Authors:** Yu Wang, Mingliang Tang, Hai Zheng, Zenghan Hu, Ya Zhong, Chuanjiang Yang

**Affiliations:** 1 College of Materials Science and Engineering, Nanjing Tech University, Nanjing 211816, China; wy17838466625@163.com (Y.W.); 18552438936@163.com (C.Y.); 2 Donghai Institute of Advanced Silicon-Based Materials, Nanjing Tech University, Nanjing 222300, China; 15996130575@163.com (H.Z.); huzenghan@163.com (Z.H.)

**Keywords:** organic bentonite, silicon-based ceramic cores, stereolithography, rheology, mechanical properties

## Abstract

With the advancement of aero-engine thrust-to-weight ratios, turbine blades now incorporate complex hollow structures fabricated using ceramic cores. The emergence of light-curing 3D printing technology for ceramic cores offers a viable solution to producing such complex structural components. To avoid the breakage of the core when removing the support after the printing of the general paste, we used a rheological additive, organic bentonite, to prepare a light-curing 3D-printed silicon-based ceramic core paste that can allow for unsupported printing. This study pursues two primary research objectives: Firstly, the effect of organic bentonite on the rheological behavior and stability properties of silicon-based ceramic was investigated. Secondly, we conducted a comprehensive analysis of how organic bentonite modification influences the performance of silicon-based ceramics. The results show that, firstly, the addition of organic bentonite dramatically improves the rheology and stability of silicon-based ceramic paste, and that the optimal content is between 1 and 2 wt.% for the best effect. Second, after the primary sintering process (1250 °C), partial bentonite can produce a small amount of cordierite phase and promote the generation of cristobalite. The room-temperature performance of the ceramic core can be improved. However, organic bentonite, after secondary sintering at 1550 °C, completely forms cordierite and reduces the amount of square quartz produced. Then, it negatively affects the high-temperature performance of the ceramic core. Therefore, when the content of organic bentonite is 1 wt.%, the ceramic paste has superior rheology and stability, making unsupported printing possible. Our study revealed an apparent porosity of 32.43%, a bulk density of 1.64 g/cm^3^, a sintering shrinkage value of 2.94%, a room-temperature flexural strength of 24.7 MPa, a high-temperature (1550 °C) flexural strength of 10.1 MPa and a high-temperature deflection of 1.24 mm, which meet the requirements of core printing.

## 1. Introduction

The global aerospace sector has witnessed intensified research on aero-engine advancement, driven by escalating demands for enhanced thrust-to-weight ratios and fuel efficiency [1,2]. Within this technological evolution, turbine blades [3,4] emerge as critical components determining engine performance characteristics. Contemporary fifth-generation engines require turbine inlet temperatures exceeding 2000 K, yet metallic alloys approach their thermal limitation thresholds (melting points ≤ 1700 K) [5,6]. This thermodynamic constraint necessitates innovative cooling strategies, mainly through complex hollow structures fabricated via ceramic core [7,8] technology. Traditional ceramic core production predominantly employs hot-press injection molding [9], a process constrained by multiple limitations, mandatory mold fabrication [10], protracted production cycles, and prohibitive costs for complex geometries [11]. As a new generation of rapid prototyping technology, 3D light-curing prototyping technology has the superior technical advantages of a high forming speed [12], superior model freedom, low material waste, and precise dimensional accuracy in terms of the finished product [13,14], without a need to make molds in advance. The model can be modified in a customized was, significantly shortening the development span of the iterative preparation cycle.

Ceramic cores are conventionally classified according to their matrix composition into four primary categories: silicon-based [15,16,17,18], aluminum-based [19,20], magnesium-based, and nano-composite systems [21]. Among these, silicon-based and aluminum-based variants dominate industrial applications due to their distinct thermomechanical properties. Silicon-based ceramic cores demonstrate superior operational characteristics in high-temperature applications, such as the following: (a) low thermal expansion coefficients (α = 4.1 × 10^−6^ K^−1^, 20–1000 °C) [22]; (b) enhanced refractoriness (>1700 °C); (c) high flexural strength; (d) chemical inertness toward superalloys under service conditions; (e) facilitated core removal via alkaline leaching. These advantages make silicon-based materials the preferred choice for aero-engine turbine blade investment casting.

The performance of ceramic paste plays a critical role in determining the quality of 3D-printed ceramic cores during the molding process [23]. Producing high-quality ceramic pastes is essential for successful ceramic core fabrication through 3D printing technology [24]. This foundation enables the production of ceramic cores characterized by high density, minimal crack formation, and a homogeneous microstructure [25,26,27]. Current research has predominantly focused on silicon-based ceramic core pastes. For instance, Jin et al. [28] systematically examined the dispersion efficiency of various dispersants in ceramic powders, subsequently optimizing paste fluidity and stability. Their work developed a silicon-based ceramic slurry with a 70 vol% solid content, demonstrating a viscosity of 10,520 mPa·s at a 12.6 s^−1^ shear rate. Sun et al. [29] conducted similar studies, showing that zirconia suspensions with specific rheological additives had viscosities reduced to 1680 mPa·s at a shear rate of 18.6 s^−1^. Similarly, Yu et al. [30] identified that nano-silica additions modified the core microstructure and enhanced rheological properties—notably, the incorporation of 2.5 wt.% nano-silica yielded a ceramic slurry viscosity of 2090 mPa·s, approaching the practical threshold for printability. Ye et al. [31] systematically investigated the effects of resin formulation, dispersant, particle size, solid content and ball milling time on the properties of SiC ceramic pastes, and produced a low-viscosity, high-solid-content ceramic paste. Wang et al. [32] analyzed the effects of modifiers and dispersants on paste properties in terms of molecular structure, and based on the fact that the better flexibility of the dispersant brought about a more pronounced change in viscosity, the dosage of the dispersant was first determined and then adjusted to obtain self-supporting pastes with 82 wt.% and 24.9 Pa·s of an ungraded distribution. Kennedy et al. [33] used oleic acid at a concentration of 0.0–0.3 wt.% to improve the rheological properties of a resin to fabricate solid cylinders and scaffolds by digital photofinish printing in a scratch-free system. These studies collectively demonstrate that modified conventional ceramic pastes can have optimized viscosities and superior rheological performance.

This study proposes a novel organobentonite-modified photopolymerizable silicon-based ceramic paste for support-free 3D printing using vat photopolymerization. The engineered slurry demonstrates superior rheology, structural stability, and controlled porosity, enabling the direct fabrication of complex ceramic cores without auxiliary supports. During printing, the paste maintains exceptional structural retention on the build platform, resisting collapse while allowing smooth blade-leveling operations. This rheological optimization ensures the dimensional fidelity of printed green bodies and facilitates the long-term storage stability of the uncured paste system.

Furthermore, systematic investigations were conducted to elucidate the correlation between organic bentonite content and the critical performance of silicon-based ceramics.

## 2. Materials and Methods

### 2.1. Raw Materials

The ceramic core slurry consisted mainly of a ceramic powder and an organic resin, while the powder was 78 wt.%. The ceramic powder part consisted of spherical silica micro powder (SiO_2_, purity 99.95%, Lianrui New Materials Co., Ltd., Lianyungang, China, particle size 6 um:0.5 um = 9:1), zirconium silicate powder (ZrSiO_4_, Purity 99%, Shijiazhuang Sizhou New Material Co., Ltd., Shijiazhuang, China), and alumina powder (Al_2_O_3_, Purity 95%, Lianrui New Materials Co., Ltd., Lianyungang, China) with a weight ratio of 16:3:1. The specific composition of ceramic powders is shown in Table 1. In addition, 0–2.5 wt.% of the total weight of the ceramic powder was added as organic bentonite (FHGEL-979, purity 99%, Zhejiang Fenghong New Material Co., Ltd., Lin’an, China). The organic resin part contained the monomer 1,6-hexanediol diacrylate (HDDA, purity 99%, Shanghai Youming Chemical Co., Ltd., Shanghai, China) as a primary crosslinker, monomer trimethylolpropane triacrylate (TMPTA, purity 99%, Shanghai Youming Chemical Co., Ltd., Shanghai, China) for enhanced network density, oligomer polyurethane acrylate (PUA, purity 99%, Shanghai Youming Chemical Co., Ltd., Shanghai, China), providing structural flexibility, and plasticizer alcohol ester XVI (TXIB, purity 99%, AVIC New Materials Co., Ltd., Bengbu, China), optimizing rheological behavior, and the ratio was 1:1:2:1. The specific composition of organic resin is shown in Table 2. The main components of ceramic pastes are listed in Table 3. Other additives include photoinitiator 2,4,6-trimethyl benzoyl-diphenylphosphine oxide (TPO, purity 99%, Guangzhou Lihou Trading Co., Ltd., Guangzhou, China, 2 wt.% resin basis), and dispersant KOS110 (0.15 wt.% of ceramic powder).

### 2.2. Paste Preparation and Printing

The ceramic powder underwent sequential pretreatment through a two-stage milling process. Initially, 200 g of ceramic powder was blended with 200 mL anhydrous ethanol, planetary-milled (240 rpm, 180 min), and then dried at 60 °C for 10 h. Subsequent high-energy milling (350 rpm, 360 min) with 200-mesh sieving and final drying (120 °C, 10 h) yielded optimized particles. Concurrently, a photocurable premix containing a photosensitive resin, an initiator, a plasticizer, and a dispersant was formulated using a speed mixer (800 rpm, 5 min). The pretreated powder was then incrementally incorporated into the premix solution through manual blending followed by vacuum homogenization (1500 rpm, 3 min), achieving homogeneous paste formation.

**Figure 1:**
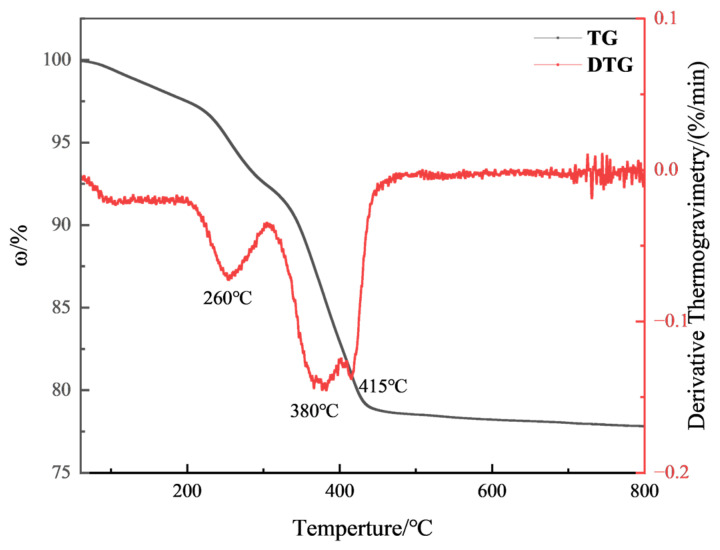
TG-DTG curves of green ceramic body.

**Figure 2:**
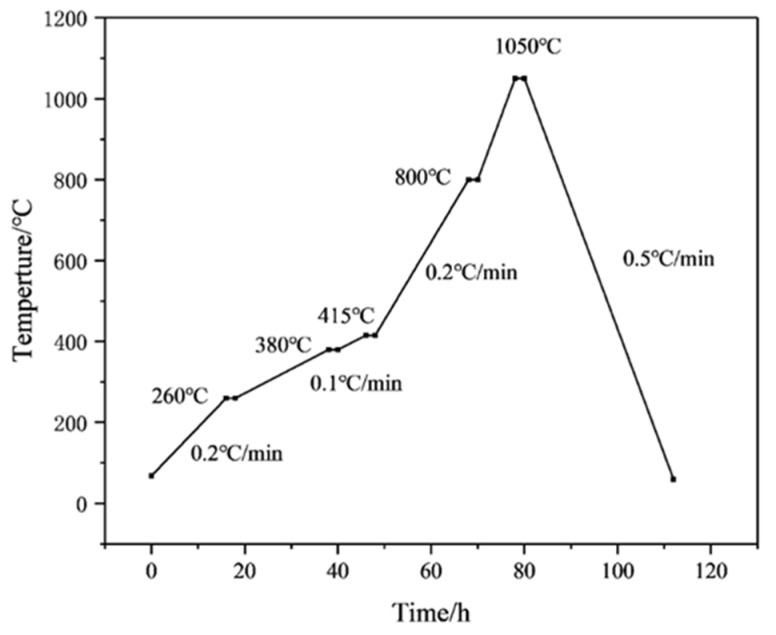
Degreasing curve of green ceramic.

**Figure 3:**
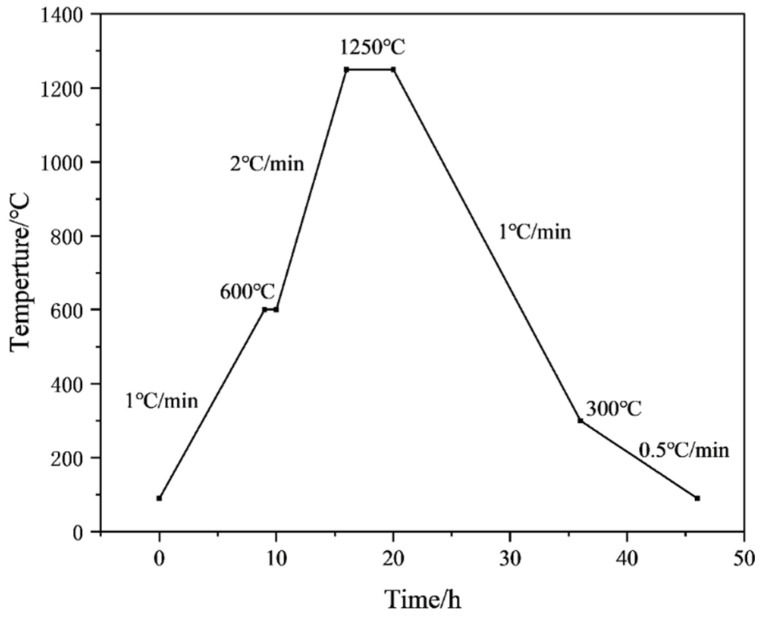
Sintering curve of green ceramic.

Then, the samples were printed with a 3D printer. The monolayer thickness of the 3D model was 50 μm, the laser power was 40 mW/cm^2^, and the laser wavelength was 355 nm. Two ceramic raw blanks, measuring 60 mm × 10 mm × 4 mm and 120 mm × 10 mm × 4 mm, were fabricated, and ceramic cores with complex structures were prepared by using an SLA-3D printer (Kangshuo Intelligent Manufacturing Co., Ltd., Suzhou, China). The blanks were immersed in a distilled water–ethanol solution, ultrasonically washed for 20–30 min to remove the residual slurry and dried. Finally, the samples were obtained after degreasing and sintering by performing thermal analysis tests on the printed blanks and then developing a degreasing and sintering curve based on the test results. A nitrogen atmosphere was used in the degreasing process and an air atmosphere was used in the sintering process. The thermal analysis test conditions were as follows: a nitrogen atmosphere, a temperature range of 25–800 °C, a holding time of 2 h, and a temperature increase rate of 2 °C/min. The test results are shown below Figure 1. The Degreasing sintering curve is also shown below Figure 2 and Figure 3.

### 2.3. Characterization

#### 2.3.1. Characterization of Paste Properties

Paste properties include rheological properties and stability. An Anton Paar MCR302 rheometer (Anton Paar, Graz, Austria) was employed to assess the rheological behavior of ceramic pastes under controlled temperature conditions (25 ± 0.5 °C). Static settling experiments characterized the stability of the ceramic paste. Briefly, 40 wt.% of ceramic paste was used as the test object, and the sample paste was put into a 50 mL test tube and left to stand for 24 h. The settling volume of the particles (V1) and the original volume of the paste (V2) were recorded individually, and V1 was used as the stability test result of the paste.

#### 2.3.2. Characterization of the Physical Properties, Mechanical Properties, and Phase Composition of Ceramic Specimens

The evaluation of ceramic core performance encompassed five critical parameters: apparent porosity, bulk density, dimensional shrinkage, and flexural strength. Phase composition analysis was conducted via X-ray diffraction (XRD, SmartLab, Longmont, CO, USA, 40 kV/30 mA, 5–90° 2θ, 0.02° step size).

A triplicate sampling strategy was implemented across all test groups to ensure statistical validity, achieving measurement reproducibility with a <5% relative standard deviation (RSD).

The apparent porosity (B, %), water absorption (W, %), and bulk density (d, g/cm^3^) of the silica-based ceramic core samples were tested. The formulas are as follows.

(1) $$B=\frac{G_{2}-G_{1}}{G_{2}-G_{3}}\times 100\%$$

(2) $$W=\frac{G_{2}-G_{1}}{G_{1}}\times 100\%$$

(3) $$d=\frac{G_{1}\times d_{water}}{G_{2}-G_{3}}$$

where $G_{1}$ is the mass after drying in the oven at 110 °C for 2 h; $G_{2}$ is the weight of the water-saturated sample in air; $G_{3}$ is the weight of the water-saturated sample in water.

The samples’ firing shrinkage (δ, %) was tested. The formula is as follows.

(4) $$\delta =\frac{L-L_{1}}{L}\times 100\%$$

where L is the length of the sample before roasting, and $L_{1}$ is the length of the sample after roasting.

The test samples’ flexural strength ($\sigma_{W}$, MPa) with silicone-based ceramic cores were determined. The formula is as follows.

(5) $$\sigma_{W}=\frac{3PL}{2bh^{2}}$$

where P is the load at the time of sample destruction; L is the span of the two pivot points; b is the width of the sample; h is the thickness of the sample.

To test the thermal deformation or high-temperature deflection (∆H, mm) of samples of silicon-based ceramic cores, the test method that should be used is the single-pivot-point method. The calculation formula is as follows.

(6) $$∆H=H_{1}-H_{2}$$

where $H_{1}$ is the height of the specimen before the test; $H_{2}$ is the height of the specimen after the test.

## 3. Results and Discussion

### 3.1. Influence of Organic Bentonite on the Rheology and Stability of Ceramic Pastes

**Figure 4:**
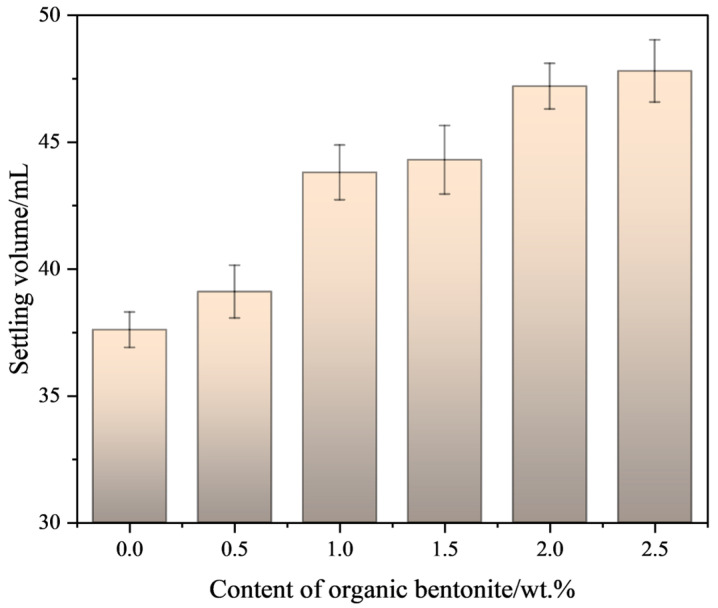
Settling volume of slurry under different contents of organic bentonite.

**Figure 5:**
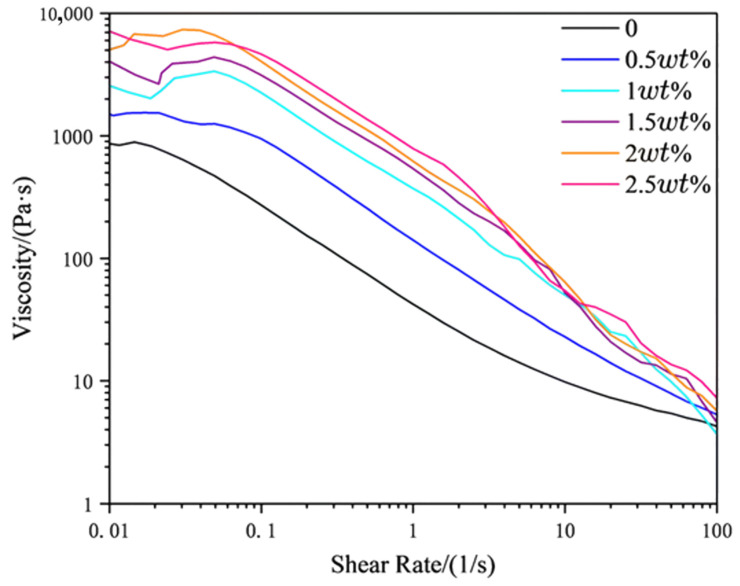
Rheological curve of slurry with different contents of organic bentonite.

A series of ceramic pastes with systematically varied organic bentonite concentrations were formulated, maintaining constant mass ratios between ceramic powder and organic resin components throughout the experimental matrix. The settling properties were tested as shown in Figure 4. The results of the rheological properties were tested as shown in Figure 5.

Organic bentonite is a kind of bentonite modified by organic ammonium. The ceramic paste prepared in this paper belongs to the oily system. Traditional bentonite has poor lipophilicity and cannot be uniformly dispersed in an oily system; however, organic ammonium-modified organic bentonite can be uniformly dispersed in an oily system.

Figure 4 shows that for a paste with a 40 wt.% solid content, after 24 h of settling, an organic bentonite content of 1 wt.% or higher improves system stability, increasing the settlement volume from 37.6 mL to 47.8 mL. If it can be ensured that a 78 wt.% solid paste does not change for a long time under normal printing conditions, it can be conclude that there is basically no resin precipitation. This is because the organic bentonite retains the structure of bentonite itself. Bentonite is mainly composed of montmorillonite; its crystal structure is 2:1-type layered silicate, and the crystal structure of montmorillonite is two layers of silica–oxygen tetrahedra sandwiched between a layer with an aluminum–oxygen octahedral composition; this structure give the bentonite a layered structure. Therefore, organic bentonite can form a specific support structure in paste systems, thus improving the stability of the paste.

The results of the rheological properties test show that the viscosity of all the samples of pastes reduced with increasing shear rates, and that all paste samples are typically non-Newtonian fluids [23,34]. The paste system without adding organic bentonite has specific thixotropic properties because the photosensitive resin contains polyurethane organic and inorganic powder, mixing easily to form specific thixotropic properties. However, in the solid content of 78 wt.% of the paste system at the shear rate of 0.01 s^−1^, similar to the stationary state. The viscosity is only 838 Pa·s, resulting in easy collapse, and it can not form a stable state. With the rise in organic bentonite content, the viscosity of the paste system basically maintains an upward trend, and the maximum it can reach is 7907 Pa·s. However, the viscosity must not be too high; otherwise, the paste may clog the squeegee during printing, impeding smooth flow and disrupting the process. Therefore, we have to choose the most suitable amount of organic bentonite to ensure that the paste in the stationary state retains the required stability in the printing process, while still being able to slide smoothly out of the squeegee. In addition, in terms of the static stabilization of ceramic pastes, at a shear rate of 0.1 s^−1^, viscosities greater than 1000 Pa·s are required for the paste to be stable and fluidity-free. According to the test results, when the shear rate is 0.1 s^−1^, the paste viscosities are 272 Pa·s, 951 Pa·s, 2255 Pa·s, 3145 Pa·s, 4068 Pa·s, and 4659 Pa·s. Therefore, static stabilization is ensured when the content of organic bentonite is greater than 1 wt.%. At a shear rate of 30 s^−1^, the viscosities of different ceramic pastes are 6.75 Pa·s, 10.46 Pa·s, 23.24 Pa·s, 16.96 Pa·s, 20.04 Pa·s, and 30.25 Pa·s. Compared to their viscosities at 0.1 s^−1^, all ceramic pastes exhibit significant viscosity reduction. The viscosity differences between 30 s^−1^ and 0.1 s^−1^ are 265.25 Pa·s, 940.54 Pa·s, 2231.76 Pa·s, 3128.04 Pa·s, 4047.96 Pa·s, and 4628.75 Pa·s. These results demonstrate that incorporating organic bentonite into ceramic pastes substantially enhances their rheological properties. When the organic bentonite content is below 2 wt.%, the improvement in rheology is more pronounced, whereas exceeding a content of 2 wt.% results in a gradual slowdown in rheological enhancement.

In the actual printing process, not only can the requirements of the ceramic paste be met, but the paste can also be easily shaken off of the scraper. Organic bentonite induces thixotropic behavior through platelet-structured networks, enhancing green body rigidity while increasing blade–paste interfacial friction. Therefore, it is necessary to investigate the static stability, blade slippage behavior, and blade adhesion characteristics of ceramic pastes. Specific results are shown in Table 4. below. The findings indicate the following: when the organic bentonite content exceeds 1 wt.%, the ceramic paste demonstrates good static stability; when the organic bentonite content is below 2 wt.%, the ceramic paste can rapidly slide off the blade; when the organic bentonite content ranges between 1 and 2 wt.%, the ceramic paste does not adhere to the blade.

Therefore, the added organic bentonite content in the range of 1~2 wt.% is suitable.

### 3.2. Influence of Organic Bentonite on the Room-Temperature Properties of Silicon-Based Ceramic Cores

**Figure 6:**
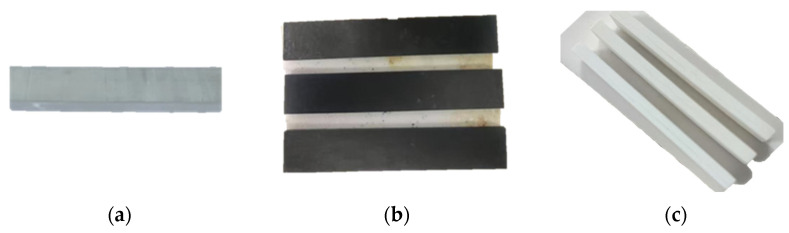
Silicon-based ceramic specimens (**a**) after printing; (**b**) after degreasing; (**c**) after sintering.

In this study, various sample pastes are printed into standard specimens by SLA light-curing 3D printers. Then, we utilize a two-step degreasing method for degreasing. The first step was to degrease the specimen in a nitrogen environment, which reduced the oxidative decomposition process of the organic resin, and only required the high-temperature decomposition of the organic resin to first remove most of the organic matter, such as O, H, N, etc., and to retain the C to form a C skeleton in the body of the billet, which not only slowed down the degreasing process and reduced the risk of cracking the billet, but also improved the strength of the billet after degreasing. After that, the degreased billet was sintered, at which point the former stage of sintering was the second step of degreasing, removing the carbon skeleton. The end stage of sintering was the real sintering densification process. Finally, the sintered specimens were tested for physical properties, mechanical properties and phase composition. Figure 6 shows the specific sample appearance of the ceramic specimen after printing, degreasing and sintering.

**Figure 7:**
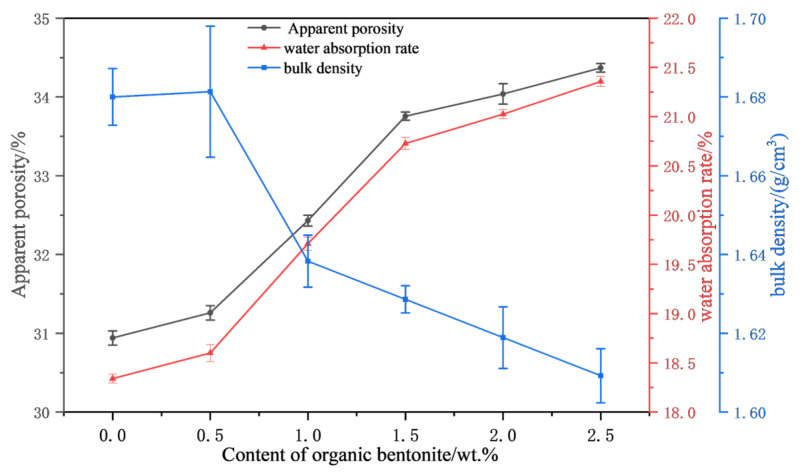
Apparent porosity, water absorption, and bulk density of ceramic test blocks under different contents of organic bentonite.

Figure 7 shows the variation in the apparent porosity, water absorption, and bulk density of ceramic samples with the amount of organic bentonite. The apparent porosity of ceramic cores is an essential indicator of the ease with which cores can be removed from the mold. Cores with higher apparent porosity are easier to remove as the casting process proceeds. As Figure 7 shows, when no organic bentonite was added, the apparent porosity of the ceramic core was 30.94%, the water absorption rate was 18.34%, and the bulk density was 1.68 g/cm^3^. When the organic bentonite content was increased to 2.5 wt.%, the apparent porosity, water absorption, and bulk density were 34.36%, 21.35%, and 1.61 g/cm^3^, respectively. The reason for this could, on the one hand, be the decomposition and volatilization of organic substances in organic bentonite at high temperatures, forming pores in the material. On the other hand, the added organic bentonite may have affected the contact and diffusion of silica particles and reduced the sintering activity.

**Figure 8:**
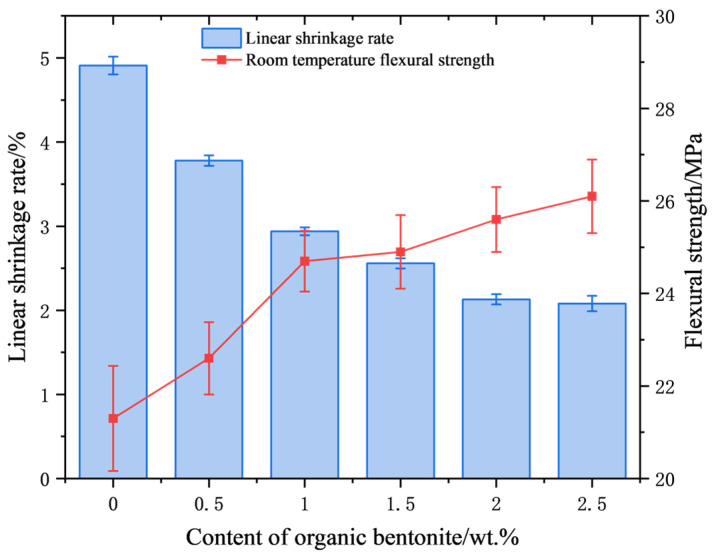
Linear shrinkage and flexural strength of ceramic samples at room temperature under different contents of organic bentonite.

**Figure 9:**
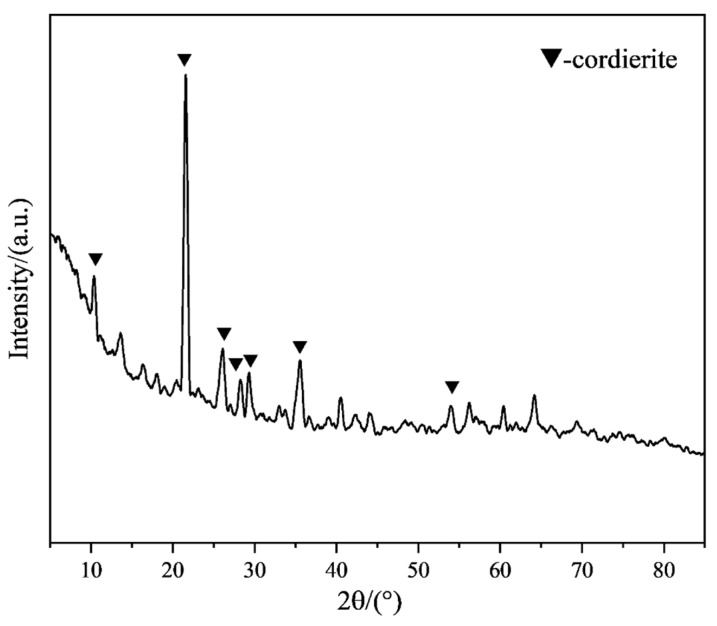
XRD pattern of organic bentonite after sintering at 1250 °C.

**Figure 10:**
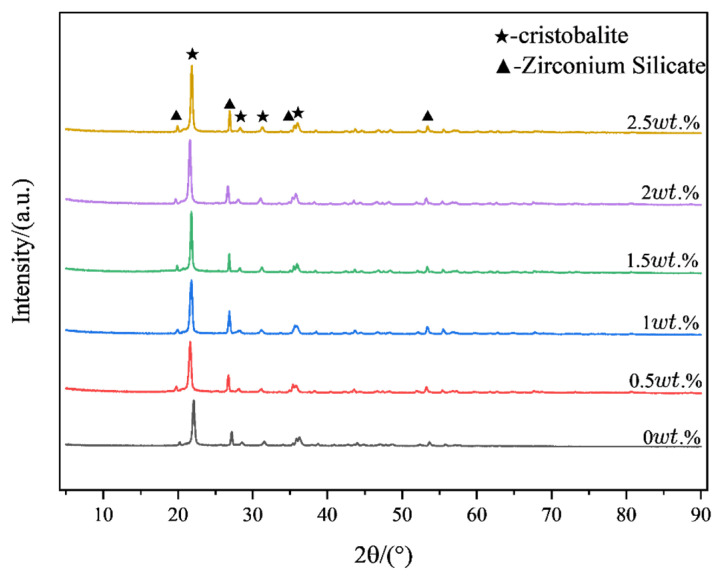
XRD patterns of ceramic test blocks with different contents of organic bentonite after sintering at 1250 °C.

Figure 8 presents the correlation between organic bentonite contents and critical sintering parameters: linear shrinkage and ambient flexural strength. Quantitative analysis reveals a dose-dependent inhibitory relationship, where increasing organic bentonite content progressively reduces dimensional contraction. The linear shrinkage of the ceramic specimens was 4.91% when no organic bentonite was added. When the content of organic bentonite increased to 2.5 wt.%, the linear shrinkage of ceramic specimens was 2.08%. The reason for this is that the layered structure of bentonite hinders the rearrangement of ceramic particles, thus reducing the shrinkage of ceramic specimens. In addition, organic bentonite can improve the flexural strength of ceramic specimens up to 26.1 MPa. The reason for this is that, on the one hand, the addition of organic bentonite can enhance the rheological properties of the ceramic paste, promote the homogeneous dispersion of the ceramic particles, and reduce agglomeration, thus improving the homogeneity of the material. On the other hand, the nanoscale montmorillonite in the organic bentonite fills in the ceramic matrix as a reinforcing phase, forming a stronger interfacial bond and improving the overall strength of the ceramic specimen. In addition, as shown in Figure 9, after sintering at 1250 °C, the contained organic bentonite forms a small amount of cordierite phase (2MgO-2Al_2_O_3_-5SiO_2_), which has high strength and toughness. The XRD patterns of ceramic specimens with different organic bentonite contents after primary sintering at 1250 °C are shown in Figure 10. The results depict that the main phases in the ceramics are cristobalite and zirconium silicate, and the peaks of cordierite phase cannot be found in the pattern because of the low content of the cordierite phase. When no organic bentonite was added, the peak intensity of cristobalite at 21.9° was 2676. When the content of organic bentonite was 2.5 wt.%, the peak intensity of cristobalite at 21.9° was 3895; the peak intensity of cristobalite in ceramics increased, and the content of cristobalite increased. This is because bentonite contains a small amount of alkaline earth metal oxides, such as MgO, which reduces the transformation temperature of cristobalite and promotes the generation of cristobalite. In silicon-based ceramics, the molten silica phase can be transformed into the cristobalite phase after sintering, which is beneficial to improving the flexural strength of the core and resistance to high-temperature creep. However, too much cristobalite will lead to an accumulation of internal stresses inside the material, causing cracks and other defects, so the content should be controlled. In this experiment, due to the small amount of organic bentonite, the generation of cristobalite had a minor impact. It did not negatively affect the flexural strength of the ceramic samples.

### 3.3. Influence of Organic Bentonite on the High-Temperature (1550 °C) Properties of Silicone-Based Ceramic Cores

**Figure 11:**
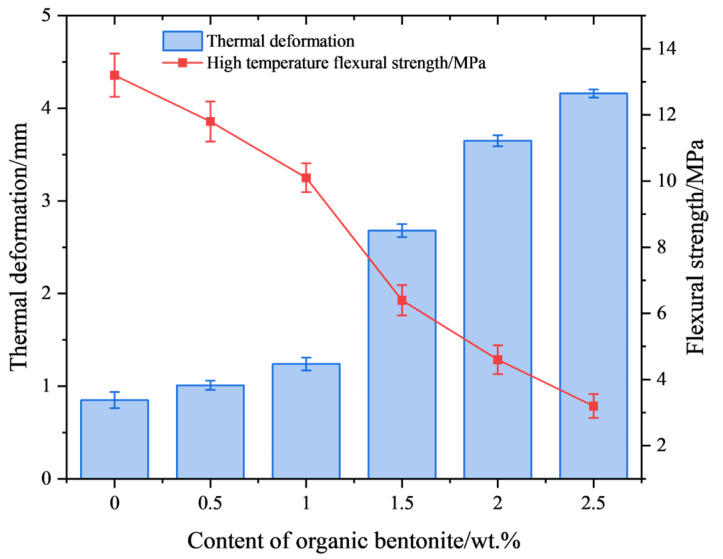
High-temperature deflection and high-temperature flexural strength of ceramic test block under different contents of organic bentonite.

**Figure 12:**
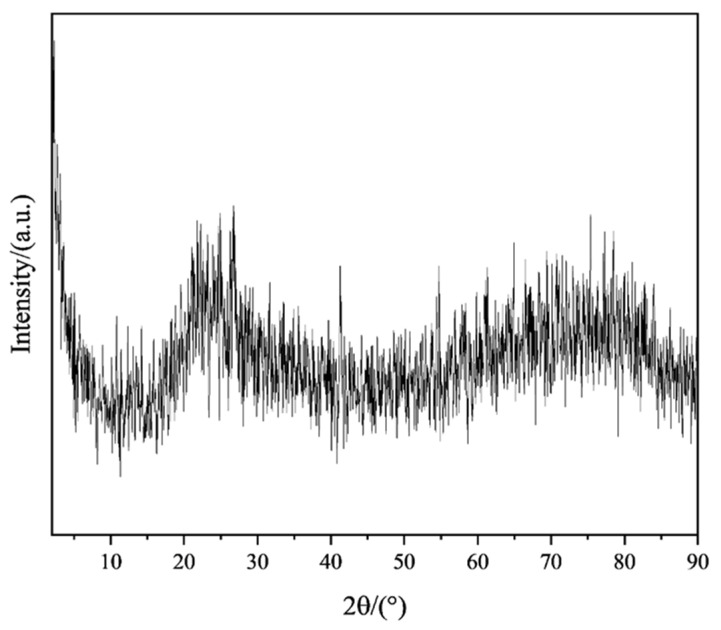
XRD pattern of organic bentonite after sintering at 1550 °C.

**Figure 13:**
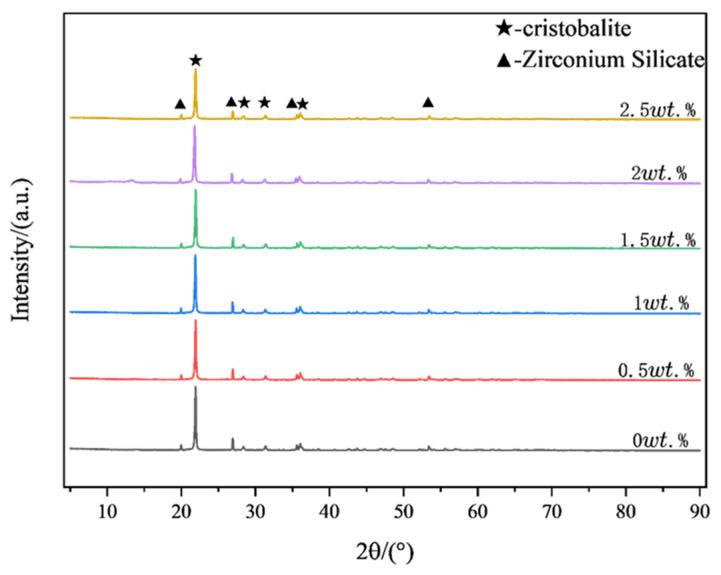
XRD patterns of ceramic test blocks with different contents of organic bentonite after secondary sintering at 1550 °C.

**Figure 14:**
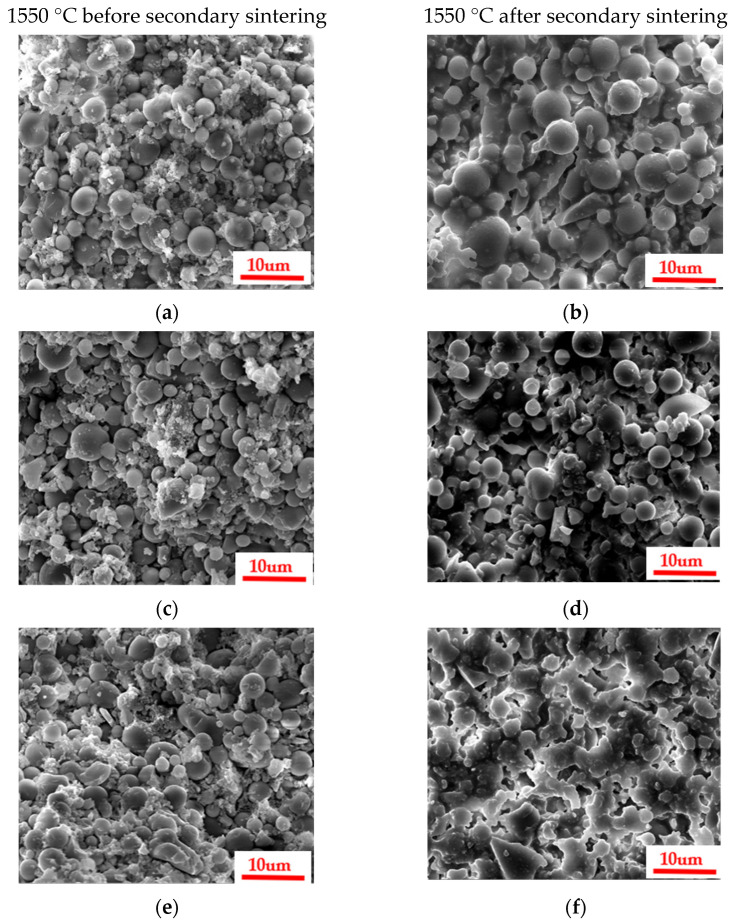

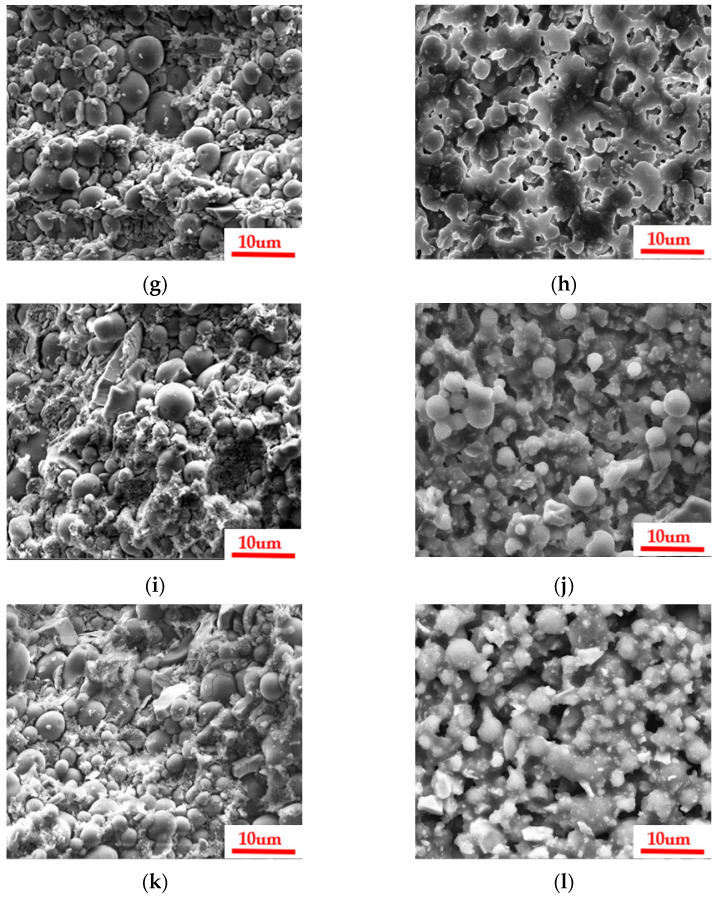
Microscopic morphology of ceramic specimens with different organic bentonite contents before and after secondary sintering at 1550 °C: (**a**,**b**), 0; (**c**,**d**), 0.5 wt.%; (**e**,**f**), 1 wt.%; (**g**,**h**), 1.5 wt.%; (**i**,**j**), 2 wt.%; (**k**,**l**), 2.5 wt.%.

The data plots of high-temperature (1550 °C) deflection and the flexural strength of ceramic specimens with different organic bentonite contents are depicted in Figure 11. From the figure, it can be seen that the addition of organic bentonite adversely affects the high-temperature performance of ceramic specimens. With the rise in the organic bentonite content, the high-temperature deflection of the ceramic specimen becomes larger, and the change is most obvious when the organic bentonite content is 1 wt.%. When the organic bentonite content is 2.5 wt.%, the high-temperature deflection increases to 4.16 mm. In addition, the increase in the content of organic bentonite ceramic specimens with high-temperature flexural strength is reduced. The reason for this is, firstly, due to the Al_2_O_3_, MgO, and SiO_2_ in the organic bentonite and the matrix material SiO_2_ forming cordierite (2MgO-2Al_2_O_3_-5SiO_2_) in the high-temperature state; secondly, it is not fully formed at 1250 °C, only fully forming at 1550 °C. In general, the transformation of montmorillonite into cordierite is followed by a volume contraction of 5–15%. Since the organic bentonite used in this paper was modified, the layer spacing between the silica–oxygen tetrahedral and aluminum-oxygen octahedral layers in the bentonite were larger than those of normal bentonite. In primary sintering (1250 °C) processes, decomposition and volatility of organic components can be observed, as can the formation of trace cordierite. Therefore, in the process of organic bentonite production, volume contraction is primarily the result of the decomposition and volatilization of organic components, present in about 60% of the product; in the secondary sintering (1550 °C) process, high-temperature stabilization of cordierite occurs, so the process of organic bentonite volume shrinkage is mainly the result of bentonite being converted into cordierite, present in 10% of the product. As a result, the resulting volume contraction caused an increase in porosity, which in turn decreased the high-temperature performance of the ceramic test pieces. Secondly, cordierite itself does not promote the high-temperature performance of ceramic specimens, but rather reduces it. Because of the thermal stability of cordierite, which has a melting point of 1460 °C, it may be partially melted or softened at temperatures close to this, which is reflected by the data on the ceramic specimen under high-temperature deflection. As shown in Figure 12, after the organic bentonite was sintered at 1550 °C, the XRD pattern only fluctuated from the baseline, and no peaks could be found, suggesting that there was no crystalline phase present. This also indicates that the resulting cordierite phase was completely melted at 1550 °C. In terms of mechanical properties, cordierite itself has low hardness and average compressive strength, and is prone to plastic deformation at high temperatures, which means that it is weak compared to both the cristobalite phase and the zirconium silicate phase of the matrix itself. Thirdly, as shown by the XRD patterns of ceramic specimens with different organic bentonite contents after secondary sintering at 1550 °C in Figure 13, in the ceramic specimens without organic bentonite, the peak intensity of cristobalite at 21.9° was 8779, and its content significantly increased relative to that in the samples after primary sintering, which was due to the fact that secondary sintering at a higher temperature is more favorable for the generation of cristobalite. However, with the increase in the content of organic bentonite in the second sintered ceramic specimens, the peak intensity of the cristobalite increasingly weakened, suggesting that the content of cristobalite was reduced, and therefore that the high-temperature performance of ceramic specimens was also reduced to a certain extent. Figure 14. illustrates the microstructural morphology of silicon-based ceramic samples with varying organic bentonite contents before and after secondary sintering at 1550 °C. As shown in the figure, prior to secondary sintering, the large spherical silica particles (D_50_ = 6 μm) in the silicon-based ceramics were densely packed, while smaller spherical silica particles (D_50_ = 0.5 μm) adhered to the surfaces of the larger particles and filled the interparticle voids. Additionally, the molten phase formed by the smaller spherical silica particles during primary sintering (1250 °C) enhanced the bonding between large and small particles, resulting in a compact structure with minimal internal pores. This microstructure contributes to the high flexural strength of the silicon-based ceramics. After secondary sintering (1550 °C), however, a noticeable increase in porosity was observed within the ceramic specimens. Furthermore, as the organic bentonite content in the silicon-based ceramics increased, both the number and diameter of pores progressively rose. When the organic bentonite content was below 1 wt.% (Figure 14b,d), small pores with diameters around 1 μm appeared. When the organic bentonite content exceeded 1 wt.%, the number of pores increased significantly, accompanied by a marked enlargement in pore size. At organic bentonite contents above 2 wt.%, large pores with diameters of approximately 8 μm were observed. This phenomenon indicates that silicon-based ceramics subjected to secondary sintering at 1550 °C exhibited significantly increased porosity and a corresponding sharp decline in flexural strength. Moreover, as the organic bentonite content in the ceramic specimens increased, their high-temperature flexural strength also gradually decreased.

In summary, the addition of organic bentonite reduces the high-temperature performance of ceramic specimens. Therefore, organic bentonite should be added as minimally as possible, but still in an amount that meets other requirements.

## 4. Conclusions

This paper investigated the influence of organic bentonite on the rheology and stability of light-curing 3D-printed silicon-based ceramic core pastes and the properties of silicon-based ceramics. The silicon-based ceramic core paste was successfully prepared without designing a support mechanism. The specific conclusions are described below.

Organic bentonite can primarily improve the rheology and stability of silicone-based ceramic pastes. To meet the printing requirements, the organic bentonite content should be maintained between 1 and 2 wt.%.

Increasing organic bentonite content enhanced the room-temperature performance of silicon-based ceramics (in terms of elevated porosity, reduced bulk density, decreased sintered linear shrinkage, and improved flexural strength). This is due to the decomposition and volatilization of organic bentonite, the sintering-induced formation of cordierite, and the promotion of cristobalite by alkaline earth metals.

When the number of organic bentonite content increases, the high-temperature performance of silica-based ceramics decreases. This is specifically manifested as a decrease in the high-temperature flexural strength and an increase in the high-temperature deflection. The specific reason is that after the second sintering at a high temperature of 1550 °C, the organic bentonite will be completely converted into a stable cordierite phase, and cordierite will be partially melted or softened at 1550 °C. Overall, cordierite’s mechanical properties are poor, and it is prone to plastic deformation. At the same time, the generation of cordierite reduces the generation of cristobalite in silica–zirconia ceramics after secondary sintering at 1550 °C.

A 1 wt.% organic bentonite content was determined to be able to deliver a silicon-based ceramic paste with optimal comprehensive performance. The specific parameters are an apparent porosity of 32.43%, a bulk density of 1.64 g/cm^3^, a sintering shrinkage value of 2.94%, a room-temperature flexural strength of 24.7 MPa, a high-temperature (1550 °C) flexural strength of 10.1 MPa and a high-temperature deflection of 1.24 mm.

## Figures and Tables

**Table 1:** Percentage of each component in ceramic powder.

Ceramic Powder Types	SiO_2_ (6 um)/wt.%	SiO_2_ (0.5 um)/wt.%	ZrSiO_4_/wt.%	Al_2_O_3_/wt.%
**Contains**	72	8	15	5

**Table 2:** Percentage of each component in organic resins.

Organic Resin Types	HDDA/wt.%	TMPTA/wt.%	PUA/wt.%	TXIB/wt.%
**Contains**	20	20	40	20

**Table 3:** Percentage of organic resin, ceramic powder and organic bentonite components in ceramic pastes of different samples.

Sample Number	Organic Resin/wt.%	Ceramic Powder/wt.%	Organic Bentonite/wt.%
No. 1	22	78	0
No. 2	22	77.61	0.39
No. 3	22	77.22	0.78
No. 4	22	76.83	1.17
No. 5	22	76.44	1.56
No. 6	22	76.05	1.95

**Table 4:** Actual state of different sample pastes.

Sample Number	Static Stabilization	Slip Situation	Adhesion Situation
No. 1	Poor	Good	Moderate
No. 2	Moderate	Good	Moderate
No. 3	Good	Good	Good
No. 4	Good	Good	Good
No. 5	Good	Moderate	Good
No. 6	Good	Poor	Poor

## Data Availability

The original contributions presented in this study are included in the article. Further inquiries can be directed to the corresponding authors.
